# A crossover randomized controlled trial examining the effects of black seed (*Nigella sativa*) supplementation on IL-1β, IL-6 and leptin, and insulin parameters in overweight and obese women

**DOI:** 10.1186/s12906-023-04226-y

**Published:** 2024-01-05

**Authors:** Elham Razmpoosh, Sara Safi, Mahta Mazaheri, Saman Khalesi, Majid Nazari, Parvin Mirmiran, Azadeh Nadjarzadeh

**Affiliations:** 1 Nutrition and Endocrine Research Center, Research Institute for Endocrine Sciences, Shahid Beheshti University of Medical Sciences, Tehran, Iran; 2 Research Center for Food Hygiene and Safety, School of Public Health, Shahid Sadoughi University of Medical Sciences, Yazd, 8915173160 Iran; 3 Faculty School of Public Health, Shahid Sadoughi University of Medical Sciences, Yazd, Iran; 4 Faculty of Medicine, Shahid Sadoughi University of Medical Sciences, Yazd, Iran; 5 Mother and Newborn, Health Research Center, Shahid Sadoughi University of Medical Sciences, Yazd, Iran; 6 Appleton Institute & School of Health, Medical and Applied Sciences, Central Queensland University, Brisbane, Australia; 7 Department of Medical Genetics, Shahid Sadoughi University of Medical Sciences, Yazd, Iran; 8 Department of Nutrition and Clinical Dietetics, Faculty of Nutrition Sciences and Food Technology Research Institute, Shahid Beheshti University of Medical Sciences, Tehran, Iran

**Keywords:** *Nigella sativa*, Nutrigenomics, IL-1β, IL-6, Leptin, Cross-over studies

## Abstract

**Background:**

*Nigella sativa* (NS) oil has been found to have advantageous benefits in the management of inflammation and obesity. This study investigated the effect of NS supplementation on blood mRNA expressions and serum levels of IL-1β, IL-6, leptin, and insulin concentrations in overweight/obese women.

**Methods:**

In a crossover design, participants were randomized to receive either NS supplements(2000 mg/day) or placebo for 2 durations(8 weeks). With between-subject and within-subject components and interactions, a repeated-measure ANOVA model was used considering the treatment, time, and the carryover effects. *Cohen’s d*(*d*) was used to measure the magnitude of the effects.

**Results:**

Forty-six eligible participants were included. NS supplementation significantly reduced the mRNA expressions(*d*=-0.68, *P* = 0.03) and serum levels of IL-1β with medium-high effect sizes(*d*=-1.6, *P* < 0.001). Significant reductions with large effect sizes were observed in the gene expression and serum levels of *IL-6*(*d*=-1.8, *d*=-0.78, respectively; *P* < 0.01) and *Leptin(d*=-1.9, *d*=-0.89, respectively; *P* < 0.01, serum leptin *P* carryover < 0.001). Despite the meaningful carryover effect for serum leptin, results remained significant following the first intervention period analysis(*P* < 0.001). A significant but low effect size decrease in serum insulin was observed(*d*=-0.3, *P* = 0.02).

**Conclusions:**

The clinical significance of present findings regarding improvements in obesity-related pro-inflammatory markers must be interpreted with caution due to some observed medium-low effect sizes.

**Trial registration:**

IRCT20180430039475N1 (Date:25/6/2018).

**Supplementary Information:**

The online version contains supplementary material available at 10.1186/s12906-023-04226-y.

## Background

Overweight and obesity are major global public health problems [1]. Nearly 40% of the world’s population is overweight or obese, which is predicted to rise steadily by 2030 [2]. Pro-inflammatory adipokine production increases as adipose tissue expands in obesity, contributing to metabolic disorders associated with obesity [3]. Obesity is linked to chronic inflammation, as well as alterations in the associated gene expression and lifestyle factors [4].

Pro-inflammatory interleukin-β (IL-1β) is a significant cytokine mainly produced by macrophages that plays a role in the development of obesity-associated insulin resistance [5]. Interleukin-6 (IL-6) is another cytokine generally referred to as an obesity-related inflammatory marker [6]. The elevated level of circulating IL-6 is correlated with adiposity and insulin resistance in humans [6]. Obese individuals are reported to have higher levels of serum IL-1β and IL-6 [7]. Adipose tissues also produce leptin, a hormone directly associated with the amount of fat stored [8]. Although it has a role in regulating food intake and body weight, leptin resistance is reported in obese individuals due to hyperinsulinemia, leading to reduced satiety and overeating [9]. Literature also suggests that leptin has pro-inflammatory properties by up-regulating cytokines such as *IL-6* [10]. *IL-1β* may also increase the expression of leptin mRNA in adipose tissue, adding to the underlying causes of obesity development [11]. The up-regulation of these pro-inflammatory factors creates a vicious circle, reducing the efficacy of obesity prevention and control efforts [11].

Dietary constituents and herbal extracts rich in phytochemicals have been recommended as affordable, effective, and safe supplemental therapies to help regulate inflammatory factors [12]. Phytochemicals are natural antioxidants with anti-inflammatory properties. The beneficial effects of phytochemicals and their derivatives on down-regulating the gene expression of pro-inflammatory and inflammatory markers have been well studied [13, 14]. *Nigella sativa* (NS), commonly known as black seed, is an herb from the Ranunculaceae family with a broad spectrum of pharmacological properties. Literature suggests that NS and its main active component, thymoquinone(TQ), have anti-hypertensive, immune-stimulatory, and weight-regulatory properties [15]. In historical medical practices, Nigella sativa (NS) oil found extensive application in addressing gastrointestinal issues like indigestion, bloating, loose stools, and inflammatory bowel conditions, alongside its utility for various other health concerns such as respiratory challenges like asthma, bronchial spasms, and congested chests. Furthermore, NS oil has been recognized for its potential as a liver tonic, diuretic, enhancer of appetite, pain-reliever, and digestive aid. It has also exhibited potential in impacting lipid metabolism, potentially assisting in the control of cholesterol levels [16].

Previous animal and experimental studies have also reported the beneficial effects of NS oil or TQ supplementation on pro-inflammatory and inflammatory markers reporting a reduction in leptin and IL-6 levels in rats with metabolic disorders [17, 18].

In humans, a few randomized controlled clinical trial (RCT) studies investigated the effect of NS oil supplements on pro-inflammatory markers and reported controversial results [19–21]. A recent study showed that a 3-month supplementation with black seed oil significantly reduced serum IL-6 and inflammatory markers in patients with chronic pulmonary disease [19]. However, no significant effects were observed on serum IL-6 levels after NS oil supplementation combined with a low-calorie diet in overweight and obese women [20]. NS oil supplementation in patients with type-2 diabetes mellitus also resulted in a significant drop in blood leptin concentration [21]. Literature on the effects of NS oil or TQ supplementation on IL-1β is scarce. Also, there is no clinical trial investigating the mechanisms of the effects of NS or TQ on pro-inflammatory cytokines in individuals with overweight or obesity. According to the evidence, the development or progression of obesity would also be independently shown by changes in peripheral blood mononuclear cell (PBMC) gene expression profiles. Therefore, this study aimed to investigate the effect of NS oil supplementation on serum concentrations and gene expression of *IL-1β, IL-6*, and *leptin* in PBMCs as well as insulin levels in a sample of overweight and obese women. The findings of this study may guide the development of more effective therapies and interventions to prevent and manage overweight and obesity in adult populations.

## Materials

### Study design and participants

This study was a crossover, double-blind, placebo-controlled, randomized clinical trial with two intervention periods of 8 weeks each and a 4-week washout period. Findings regarding the effects of *Nigella sativa* on major adipogenesis-related parameters have been published previously [22]. The trial was designed following the Consolidated Standards of Reporting Trials (CONSORT) guidelines, revised for randomized crossover trials [23]. Individuals were recruited from the obesity clinic at Shahid Sadoughi University of Medical Sciences, Yazd, Iran. Otherwise healthy women aged 25 to 55 with a body mass index (BMI) between 25 and 35 kg/m^2^ were eligible to participate. Participants were not included if they had comorbidities conditions such as type-2 diabetes mellitus, cardiovascular diseases, hyperlipidemia, pancreatic or hepatic disorders, thyroid or renal diseases, were pregnant or breastfeeding, or smoked or consumed alcohol. Those with a history of surgery, allergy, or cancer, as well as those taking obesity medications, nutritional supplements, or participating in a specific weight-loss program for at least six months prior to the study’s start date, were also excluded.

An independent researcher blinded to the study randomized participants into two groups (1:1) of NS and placebo using block randomization and computer-generated random numbers stratified based on age. Concealment was performed using sealed envelopes. Literature suggests at least 1000 mg/day of NS oil supplementation is required to observe significant changes in leptin concentration [21], and that a daily intake of 2000 mg of NS is well-tolerated with no adverse reactions [24]. Participants were given either 2000 mg/day of NS oil supplement (two capsules of 1000 mg/day oil each) or two capsules/day of a paraffin oil placebo (2000 mg in each capsule). *Nigella sativa* and placebo capsules were provided by Barij Essence Pharmaceutical Company (Kashan, Iran). The NS oil was extracted from the Ranunculaceae family grown in Iran and processed using the cold-press method. TQ was the most active component in each 1000 mg of black seed oil capsule, accounting for 1.1% of the total. The intervention and placebo capsules looked and tasted the same, and the bottles were labelled with unique codes. Additional file 1 presents the results of the gas chromatography-mass spectrometry (GC/MS) test on each 50 g of NS. Participants were instructed to consume one capsule before lunch and another before dinner. They were not allowed to alter their usual physical activities. Regular drugs, such as lipid-lowering agents or blood pressure meds, were permitted as long as the dosages were maintained during the trial. A four-week washout period was selected based on the literature to cover at least five times the half-life of the treatment intervention to eliminate the carryover effect [25]. The elimination half-life of oral administration of TQ, as the main constituent of NS, is reported to be around 275 min [26]. The washout time was extended to four weeks in this study to factor in the maximum elimination of the carryover effects. All participants and researchers were blinded to the study until the final analysis.

Individuals were also provided with an individualized diet plan without any calorie deficit to control for energy and macronutrient intakes during the study. The diet regimen was designed by a certified nutritionist based on every individual’s body weight at the start of each intervention phase. The required energy was first estimated, and then a dietary plan with portions from each food group was designed to meet the macronutrient distribution, which was 55% carbohydrates, 30% fat, and 1.2 g of protein per kg of body weight. During each intervention session, a certified dietitian monitored participant compliance with dietary intakes in both groups (at weeks 4, and 8). Participants were required to return the empty bottles or any remaining supplements at the end of each intervention period. Compliance with the intervention was also monitored three times per week through phone and face-to-face interviews, and any adverse reactions were recorded.

Sample size was calculated using the formula suggested for crossover studies [27]; after considering a non-inferiority margin of 0.05, a significance level of 0.05, a power of 80%, and anticipating a 15% dropout rate, a total of 40 participants were required for this crossover study.

### Anthropometric, dietary intake, and physical activity assessments

Weight (to the nearest 0.1 kg precision), height (to the nearest 0.1 cm precision), and body composition of individuals in light clothing and an overnight fasted state were measured using a body analyzer (In-body, 770, USA) machine. BMI was computed by dividing weight (kg) by height (m^2^). Every participant’s dietary intake was measured twice during the study using 3-day food diaries (once at the start of each intervention period) and then converted to g/day using home measurements [28]. Physical activity level was assessed using a validated questionnaire based on metabolic equivalent [29]. The questionnaire comprises nine different types of physical activity, each with different levels of intensity. The sum of the average hours spent on one day for each activity depending on the quantity of MET equivalent was used to compute the overall number of MET hours per day (h/day).

### Biochemical assessment

After a 12-h overnight fast, a venous blood sample (12 ml) was collected from participants at the beginning and end of each intervention period. Blood samples were then centrifuged for 10 min (3,000 g, at room temperature; Eppendorf AG, Hamburg), and serum samples were separated and stored at − 70°^C^. The enzyme-linked immunoassay (ELISA) method was used to assess serum levels of IL-1β, IL-6, leptin and insulin (Thermo Fisher Co., USA; inter and intra-assay coefficients of variations were < 10%).

### RNA extraction and real-time polymerase chain reaction (RT-PCR)

Literature suggests that PBMCs reflect the effects of dietary modifications at the gene expression level [30]. As reported in the GETx portal (https://gtexportal.org), *IL-1β, IL-6* and *leptin* genes have been identified in blood by RNA sequencing (Additional file 2). The standard Ficoll method was performed to isolate PBMCs from whole blood. Total RNA was extracted immediately using the Qiagen RNA purification kit (Qiagen Co., Germany, Cat No. 74,104). The spectrophotometer (NanoDrop, Thermo Scientific) was used for assessing the quality and purity (260/280 nm ratio between 1.8 and 2.2) of the extracted RNA. Then, total mRNA was reverse-transcribed into cDNA using a cDNA synthesis kit (Thermo Fisher Co., USA, Cat No. RR037).

The expressions of *IL-1β, IL-6* and *Leptin* genes were quantified using real-time polymerase chain reaction (RT-PCR) along with the SYBR Green method (Takara Bio, Inc., Japan). To verify the product’s specificity, melting curve analyses were performed at the end of every run. Primers for RT-PCR were designed using Primer Blast, Oligocalc, and Gene-runner 5.0.99 (Table 1). All quantifications were normalized to glyceraldehyde phosphate dehydrogenase (GAPDH) as the housekeeping gene. The efficacy of PCR was examined using LinRegPCR software [31]. Finally, cycle threshold (Ct) values were obtained, and fold changes (FD) were calculated through ∆∆Ct method [32].

**Table 1:** Real-time PCR primer sequences

	Forward	Reverse
*IL-1β*	TGATGGCTTATTACAGTGGCAAT	AAGCCCTTGCTGTAGTGGTG
*IL-6*	CCCTGAGAAAGGAGACATG	TGATTTTCACCAGGCAAGTC
*Leptin*	TCCCCTCTTGACCCATCTC	GGGAACCTTGTTCTGGTCAT
*GAPDH*	ACAACTTTGGTATCGTGGAAGG	GCCATCACGCCACAGTTTC

**Abbreviations**: PCR, polymerase chain reaction; *IL-1β*, interleukin-1 beta; *IL-6*, interleukin-6; *GAPDH*, glyceraldehyde-3-phosphate dehydrogenase

### Statistical analysis

Means and confidence intervals (CI) were reported for continuous variables. Differences in baseline characteristics and outcomes of interest between groups were evaluated using independent samples t-test. Skewness and Kurtosis tests were performed to assess the normality of data. If any baseline variables did not have a normal distribution (*P* Skewness < 0.05), the BoxCox transformation was used before the final analysis. The final analysis was based on the intention-to-treat (ITT) method. All randomized individuals for whom relative data were available for at least the first period of intervention were considered as the ITT population. Specific statistical operations are required for the cross-over design. With between-subject and within-subject components and interactions, a repeated-measure ANOVA model was used considering the effect of treatment, time, and interaction between them, which was called as the carryover effect. If the carryover effect was found to be significant (residual *P* < 0.05), the results of the first intervention period were analyzed using ANCOVA test. Additionally, The magnitude of the treatment effects was then assessed using a one-sample t-test (two-sided, alpha level of 0.05), estimating *Cohen’s d*, for all participants with complete data, evaluating differences in outcome while on the NS oil period vs. changes in outcome when on the placebo period. The effect size was defined as small (*Cohen’s d* = 0.2), medium (*Cohen’s d* = 0.5), and large (*Cohen’s d* = 0.8). The *Cohen’s d* value of zero indicates that there are no differences between the means of the two comparative groups, and 50% of the observations in the control group are located below the mean of the experimental group. The *Cohen’s d* values of 0.2, 0.5, and 0.8 are located at the 58th, 69th, and 79th percentiles of the distribution of the control group, respectively [33]. A *P* < 0.05 was considered statistically significant. All analyses were performed using SPSS version 21.

### Ethical approval

The protocol of the study was registered at the Iranian Registry of Clinical Trials (registration no. IRCT20180430039475N1, date:25/6/2018) [34]. Ethical approval was obtained from the Medical Ethics Committee of Shahid Sadoughi University of Medical Sciences, Yazd, Iran (approval no. IR.SSU.SPH. REC.1397.006) and the Committee of Ethics of Shahid Beheshti University of Medical Sciences (approval no. IR.SBMU.ENDOCRINE.REC.1400.084). All methods were performed in accordance with the Declaration of Helsinki. Participants were recruited in April 2019. They were provided with an information sheet and asked to complete an informed consent form prior to the study. Informed consent was obtained from all participants.

## Results

**Fig. 1:**
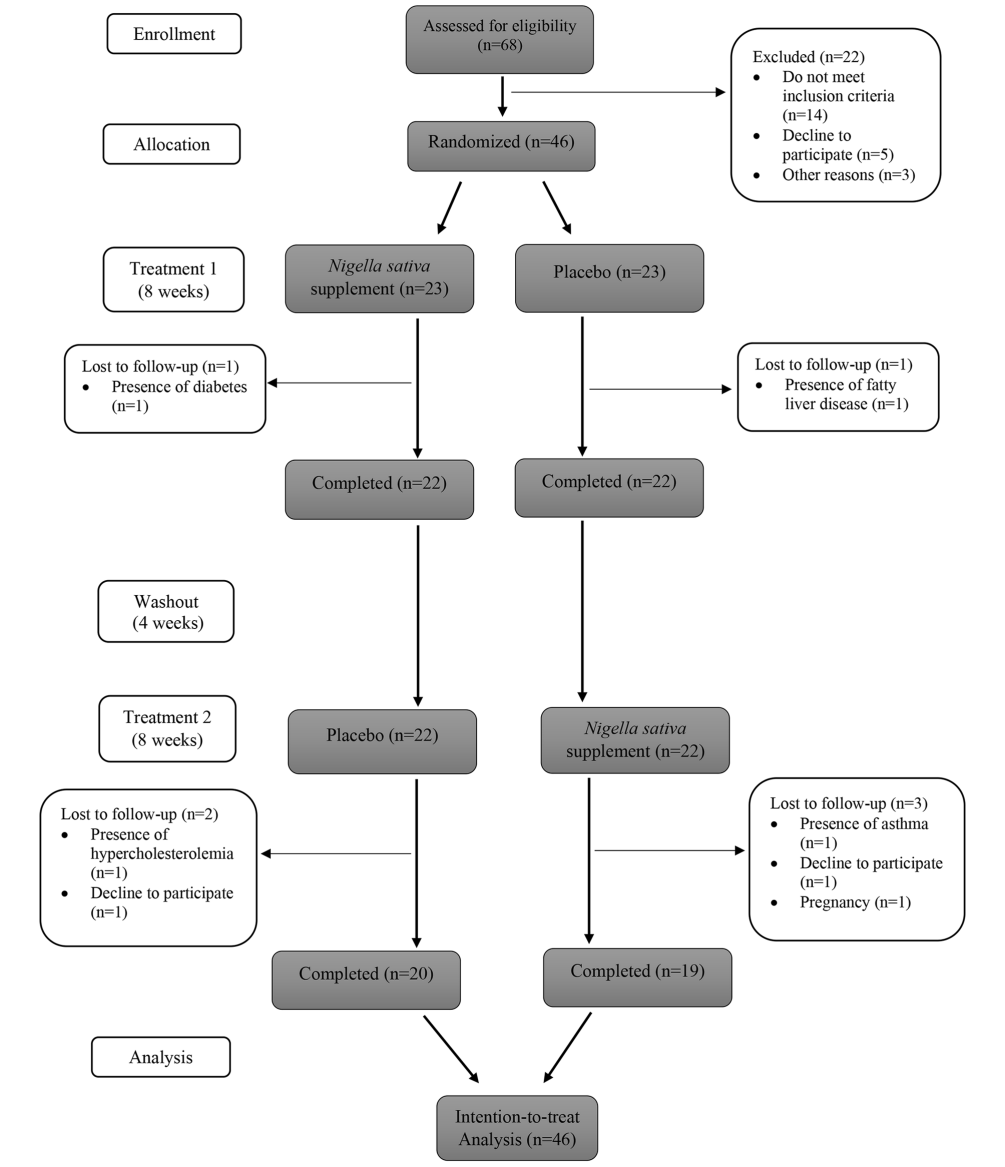
Participant flow diagram

Forty-six eligible women were included. A total of seven participants were lost to follow-up due to pregnancy (n = 1), a new diagnosis of disease (n = 4), and personal reasons (n = 2) (Fig. 1).

The baseline characteristics of participants are summarized in Table 2. The mean ± SD age, height, and BMI of participants were 36 ± 10 years, 159 ± 6.4 cm, and 32 ± 5 kg/m^2^, respectively. Baseline characteristics of physical activity status, dietary intake, medication, and serum biomarkers of IL-1β, IL-6, and leptin were not significantly different between the NS and placebo groups. Therefore, pooled baseline measures were used for the final analyses. No severe adverse reactions were reported. There was a significant decrease in BMI following the NS supplementation [mean change (SD)=-0.34 (0.62) kg/m^2^; the mean (SD) BMI was 31.02 (5.17) kg/m^2^ for the intervention period and 31.36 (5.30) kg/m^2^ for the placebo period; *P* effect = 0.004; *P* carryover effect = 0.09].

**Table 2:** Participants’ baseline characteristics

Variable	Overall (n = 46)	Group A ^a^ (n = 23)	Group B ^b^ (n = 23)	Baseline *P* value ^c^
Age (years) ^d^	36.50 (9.40)	37.70 (10.91)	34.60 (9.60)	0.35
Height (cm) ^d^	158.74 (6.61)	156.50 (4.51)	159.60 (7.20)	0.08
Body Weight (kg) ^d^	76.80 (13.90)	76.60 (13.90)	79.93 (14.8)	0.47
BMI (kg/m^2^) ^d^	31.26 (4.80)	31.15 (4.13)	31.40 (5.50)	0.91
Physical activity (MET- h/day) ^d^	25.72 (3.25)	25.91 (3.10)	25.54 (3.90)	0.67
Menopause status, (perimenopause) *n* (%)	8 (17.17)	3 (13.10)	5 (20.90)	0.48
Mild fatty liver, *n* (%)	17 (36.17)	8 (34.78)	9 (37.50)	0.51
High normal blood pressure, *n* (%)	7 (14.90)	2 (8.70)	5 (20.85)	0.25
Family history of obesity, *n* (%)	10 (25.64)	5 (26.31)	5 (25.10)	0.31
**Dietary intakes** ^d^				
Energy intake (kcal)	1780.34 (190.54)	1765.86 (212.82)	1794.25 (169.56)	0.61
Carbohydrate (gr)	222.14 (34.153)	217.25 (31.89)	226.82 (37.10)	0.35
Protein (gr)	50.90 (7.80)	52.68 (8.21)	49.21 (7.30)	0.13
Fat (gr)	79.30 (12.57)	79.70 (12.30)	78.9 (13.10)	0.84
Fiber (gr)	13.11 (2.80)	12.50 (2.95)	13.85 (2.51)	0.11
Cholesterol (mg)	144.98 (862.70)	162.43 (93.54)	128.26 (78.80)	0.18
PUFA (gr)	25.43 (5.10)	25.76 (4.74)	25.11 (5.40)	0.67
MUFA (gr)	32.20 (6.50)	31.33 (6.21)	33.10 (6.75)	0.37
SFA (gr)	14.90 (2.80)	15.26 (2.25)	14.64 (3.31)	0.51
Vitamin E (α-tocopherol) (IU)	26.01 (5.60)	25.93 (5.30)	26.11 (5.96)	0.91
Vitamin C (mg)	59.95 (24.38)	56.71 (20.61)	63.14 (27.59)	0.38
Vitamin D (μg)	3.80 (2.70)	4.10 (2.80)	3.50 (2.50)	0.48
**Medication use**				
Statins, *n* (%)	10 (21.27)	5 (21.73)	5 (20.84)	0.31
Blood pressure lowering drugs, *n* (%)	3 (6.38)	0 (0.0)	3 (12.50)	
Both drugs, *n* (%)	6 (12.78)	3 (13.10)	3 (15.50)	
No drugs, *n* (%)	20 (421.55)	11 (47.82)	9 (37.50)	
**Serum Biomarkers** ^d^				
IL-1β (pg/ml)	2.95 (1.42)	2.75 (1.45)	3.00 (1.40)	0.42
IL-6 (pg/ml)	2.85 (1.52)	2.70 (1.50)	1.85 (0.50)	0.76
Leptin (ng/ml)	41.30 (6.80)	41.90 (6.22)	40.80 (7.31)	0.63
Insulin (μU/m)	6.42 (0.79)	6.18 (0.93)	6.29 (0.87)	0.56

^a^ Participants who started with NS supplementation and then went on to placebo

^b^ Participants who started with placebo and then went on to NS supplementation

^c^ Independent *t* test was used to compare between-group measurements

^d^ Data are shown in Mean ± SD

BMI, body mass index; MET, metabolic equivalent; PUFA, poly-unsaturated fatty acids; MUFA, mono-unsaturated fatty acids; SFA, saturated fatty acids; *IL-1β*, interleukin-1 beta; *IL-6*, interleukin-6.

### Effects of ***Nigella sativa*** (black seed) oil supplement on mRNA gene expressions of ***IL-1β, IL-6***, and ***Leptin***

**Fig. 2:**
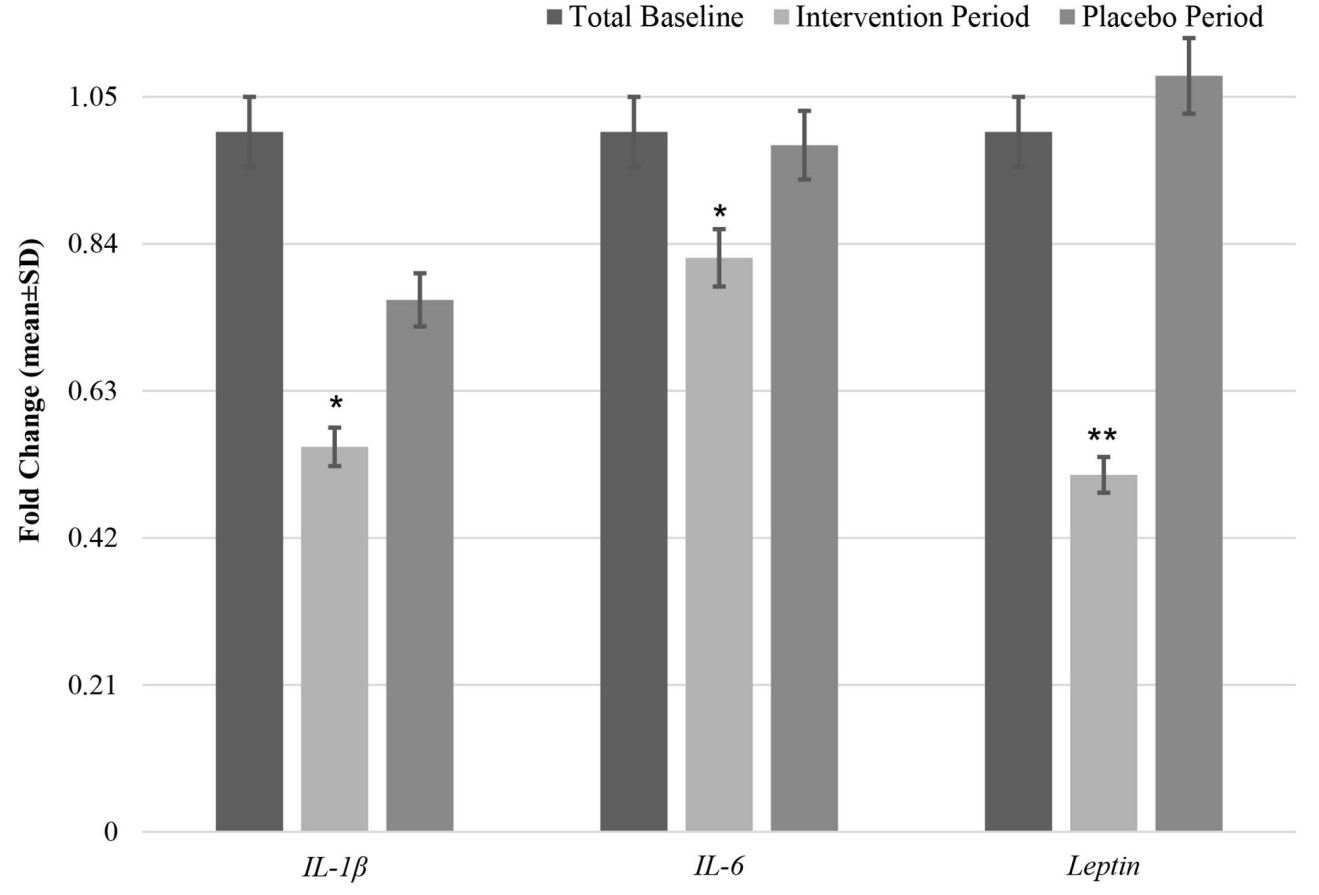
Fold changes (means ± SDs) in mRNA expression levels of *IL-1β*, *IL-6* and *Leptin* in PBMCs during NS intervention period *versus* placebo period among women with overweight and obesity (n = 46) A repeated-measure ANOVA model was used considering the effects of treatment, time, and interaction (carryover effect) * *P* value < 0.05; ** *P* value < 0.001 Abbreviations: NS, *Nigella Sativa; IL-1β*, interleukin-1 beta; *IL-6*, interleukin-6

Figure 2 presents the changes in mRNA expression levels of *IL-1β*, *IL-6* and *leptin* following NS or placebo supplementation. After accounting for the treatment, period, and carryover effects, NS supplementation led to a 0.21 fold change (FC) decrease in *IL-1β* gene expression with a medium effect (*P* carryover effect = 0.16 and *P* effect = 0.03, *Cohen’s d*=-0.68), a 0.17-FC decrease in the gene expression levels of *IL-6* with a large effect size (*P* carryover effect = 0.11; *P* effect = 0.003, *Cohen’s d*=-1.8), and a 0.57-FC decrease in *leptin* mRNA gene expression with a relatively large effect size (*P* carryover effect = 0.67; *P* effect < 0.001, *Cohen’s d*=-1.9) compared to the placebo.

### Changes in serum concentrations of IL-1β, IL-6, leptin and insulin

Consistent with the findings from RT-PCR, NS supplementation resulted in a significant reduction in serum concentrations of IL-1β with a large effect size (*P* effect < 0.001, *P* carryover effect = 0.31, *Cohen’s d*=-1.6), IL-6 with a medium-large effect size (*P* carryover effect = 0.08; *P* effect = 0.002 *Cohen’s d*=-0.78), and serum leptin concentration with a large effect size (*P* carryover effect < 0.001; *P* effect = 0.008, *Cohen’s d*=-0.89) compared to placebo (Table 3). Although a significant carryover effect was seen in the analysis of leptin concentration, a significantly lower level of leptin was still observed in the NS group compared to the placebo group at the end of the first treatment period (28.34 ± 6.5 ng/ml vs. 39.65 ± 7.2 pg/ml, respectively, *P* < 0.001). Results also showed a significant reduction but with a low effect size in serum fasting insulin levels following NS supplementation (*P* effect = 0.02, *P* carryover effect = 0.81, *Cohen’s d*=-0.3).

**Table 3:** Final changes in mRNA expression levels in PBMCs and serum concentrations of IL-1β, IL-6 and leptin (and serum insulin) in NS intervention period and placebo period among women with overweight and obesity (n = 46) ^**a**^

Variable ^b^	Black seed oil baseline	Black Seed oil final	Δ Black seed oil	Placebo baseline	Placebo final	Δ Placebo	Treatment effect	*Cohen’s d* ^*c*^	*P* treatment effect	*P* carryover effect
*IL-1β* (fold change)	1	0.55 (0.42 to 0.68)	-0.45 (-0.58 to -0.31)	1	0.76 (0.63 to 0.90)	-0.24 (-0.37 to -0.90)	-0.21 (-1.15 to -0.06)	0.68	0.03	0.16
*IL-6* (fold change)	1	0.82 (0.73 to 0.90)	-0.20 (-0.26 to 0.05)	1	0.98 (0.92 to 1.03)	-0.01 (-0.07 to 0.05)	-0.17 (-0.28 to -0.60)	1.80	0.003	0.11
*Leptin* (fold change)	1	0.51 (0.44 to 0.57)	-0.49 (-0.55 to 0.43)	1	1.08 (1.01 to 1.16)	0.08 (0.02 to 0.10)	-0.57 (-0.68 to -0.47)	1.90	< 0.001	0.67
IL-1β (pg/ml)	2.77 (2.36 to 3.17)	2.36 (2.01 to 2.71)	-0.40 (-0.54 to -0.26)	2.79 (2.40 to 3.17)	2.88 (2.50 to 3.28)	0.10 (-0.14 to 0.34)	− 0.50 (-1.30 to -0.10)	1.60	< 0.001	0.31
IL-6 (pg/ml)	2.83 (2.36 to 3.20)	2.58 (2.12 to 3.03)	-0.25 (-0.37 to -0.13)	2.67 (2.26 to 3.07)	2.63 (2.23 to 3.01)	-0.04 (-0.09 to 0.02)	-0.22 (-1.20 to -0.10)	0.78	0.002	0.08
Leptin (ng/ml)	42.32 (40.75 to 43.90)	33.78 (31.61 to 35.90)	-8.44 (-10.46 to -6.41)	34.69 (32.60 to 36.78)	29.38 (26.41 to 32.34)	-5.36 (-7.17 to -3.56)	-3.10 (-13.40 to -0.06)	0.89	0.008	< 0.001
Insulin (μU/m)	6.32 (6.07 to 6.57)	5.62 (5.43 to 5.81)	-0.70 (-0.87 to -0.51)	6.30 (6.04 to 6.57)	5.87 (5.60 to 6.12)	-0.43 (-6.75 to -0.31)	-0.27 (-3.48 to -0.05)	0.30	0.02	0.81

^a^ A repeated-measure ANOVA model considering the effect of treatment, time, and interaction (carryover effect) was used for final analyses

^b^ Data are shown in mean (CI)

^c^ The *Cohen’s d* values of 0.2, 0.5 and 0.8 locate at 58th, 69th and 79th percentile of the distribution of the control group, respectively

NS, *Nigella Sativa; IL-1β*, interleukin-1 beta; *IL-6*, interleukin-6

## Discussion

This study meticulously investigated the effect of 2000 mg/day NS oil supplementation on blood mRNA gene expressions and serum concentrations of major obesity-related pro-inflammatory factors in overweight and obese women. Our findings suggested a significant down-regulation in the mRNA expression of *IL-1β, IL-6*, and *Leptin*. Similarly, significant reductions in the serum levels of IL-1β, IL-6, leptin, and insulin were observed following NS supplementation. These findings align with existing literature that suggests the beneficial effect of NS oil supplementation on leptin levels in patients with type-2 diabetes [21] and patients with non-alcoholic fatty liver disease [35]. Notably, the substantial reduction in serum leptin levels following NS supplementation holds clinical significance. A previous prospective RCT demonstrated that a reduction of 3.3 ng/mL in leptin levels over one month is a strong predictor of long-term weight loss in adults with overweight and obesity [36]. Our study recorded a mean decrease of 3.1 ng/mL after NS supplementation.

The observed significant reductions in blood insulin levels among overweight and obese women echo findings from a study by Mahdavi et al., where NS oil supplementation in combination with a low-calorie diet led to similar outcomes [20]. However, this prior study did not report significant changes in IL-6 measures. Discrepancies in results could arise from variations in study design, sample size, population characteristics, and intervention specifics such as types and doses of NS supplementation. It’s worth mentioning that follow-up studies have associated a median reduction of 0.16 pg/ml in IL-6 levels with a reduced hazard ratio of 1.44 in cardiovascular diseases [37] and lower adiposity measurements [38]. In line with this, our research showcased a considerable decrease in IL-6 levels. Furthermore, the observed significant reductions with comparatively large effect sizes in PBMC gene expressions of IL-6, IL-1β, and leptin suggest the potential for controlled inflammation in obesity after NS oil supplementation. These results resonate with evidence indicating that alterations in PBMC gene expression profiles are independently related to obesity’s onset or progression [4]. Consequently, the significant reductions in PBMC gene expressions of *IL-6*, *IL-1β*, and *leptin*, marked by their comparatively large effect sizes, collectively point toward a potential state of controlled inflammation in the realm of obesity subsequent to NS oil supplementation.

A clear connection exists between cholesterol levels and pro-inflammatory cytokines, such as IL-1β and IL-6, particularly among participants dealing with overweight and obesity [39]. Excess body fat, especially around organs, triggers ongoing inflammation, with adipose tissue releasing these cytokines. This inflammatory state disrupts metabolism, leading to lipid imbalances like elevated low-density lipoprotein cholesterol (LDL-C) and triglycerides, alongside lowered high-density lipoprotein cholesterol (HDL-C). Raised levels of these proinflammatory cytokines can worsen inflammation, accelerating the progression of atherosclerosis and potentially heightening the risk of cardiovascular events [39, 40]. In light of this, our study demonstrated reduced cytokine levels following NS interventions. This suggests that such interventions might have the potential to modify cytokine levels, ease inflammation, and ultimately lower the risk of future cardiovascular diseases, alongside promoting other aspects of a healthy lifestyle.

The plausible mechanism behind the anti-inflammatory effects of NS supplements lies in its long-chain unsaturated fatty acids, bioactive substances, and the regulation of *IL-1*, *IL-6*, and *leptin* gene expressions as pro-inflammatory markers [15]. This aligns with the inhibition of NF-κB, TNF-α, and IL-1 receptor-associated kinase (IRAK-1) by NS or its active component TQ, leading to the suppression of the mitogen-activated protein kinase (MAPK) pathway and down-regulation of gene expressions of pro-inflammatory markers [41]. These insights could also offer an explanation for the observed beneficial changes in BMI post-NS supplementation. Furthermore, the interplay between weight gain, inflammation, and NS’s potential role in expediting weight loss underscores the multifaceted approach NS might offer in obesity management [3]. Nonetheless, it’s noteworthy that the observed final mean changes in insulin levels among overweight and obese women in our study exhibited a relatively modest effect size. Therefore, it is crucial to approach the interpretation of these results, especially concerning the insulin status and the potential beneficial role of NS in managing insulin resistance in obesity, with careful consideration.

We recognize the importance of including obesity-related stress markers in our analysis to provide a more holistic view of our results. Our study concentrated on some of the most vital obesity stress markers, such as leptin and IL-6, which represent crucial facets of the intricate obesity-associated stress network. Leptin’s role beyond appetite regulation involves signaling potential resistance patterns in obesity. Similarly, IL-6, a key cytokine, has implications in inflammation and metabolic disturbances [42]. However, it is important to acknowledge that these markers are just a subset of the complex physiological responses engendered by obesity. Exploring stress markers like adrenaline and cortisol could yield insights into the broader stress adaptation mechanisms triggered by obesity.

This study has several strengths. To our knowledge, no human studies have assessed the effect of NS supplements on gene expression of pro-inflammatory cytokines, especially in overweight and obese women. Using a crossover design with a long carryover elimination period and effect size estimations improved the validity of the results observed [43]. Also, because the NS supplementation used in this study was purely NS oil extract, the effect observed can be attributed to NS alone, as compared to previous studies that investigated the effects of NS extracts combined with other substances such as honey [44] or herbal extracts [45]. To avoid the heterogeneity that could arise from including other groups (such as male populations), this study exclusively included overweight or obese women. Finally, due to the potential confounding effects of dietary consumption, all research participants were given a customized diet plan that did not include any calorie restrictions.

However, this study also has some limitations. While a significant reduction in *leptin* mRNA expression was observed following the two intervention periods of 8 weeks, the relative decrease in serum leptin concentration can only be attributed to the first treatment period, due to the significant carryover effect observed. This carryover effect could be due to the rapid short-term changes in serum leptin levels [46]. Moreover, despite the significant reduction in serum insulin, the effect size was estimated to be small which may limit the interpretation of results for the effect of NS on serum insulin levels or the status of insulin resistance. Further assessments may also be needed to accurately measure the half-life time of NS oil supplements. In this study, the blood or urine concentration of TQ, metabolites of NS, or other pro-inflammatory markers were not measured. This could help providing a better understanding of the anti-inflammatory effects of NS oil supplementation. In addition, limiting the study participants to women to reduce the gender bias limits the generalization of the findings as females may react differently to NS bioactive compounds than males [47].

## Conclusions

In conclusion, our findings showed that daily supplementation of 2000 mg of NS oil after 2 periods of 8 weeks resulted in significant reductions in blood mRNA expression levels of obesity-related pro-inflammatory genes, including *IL-6, IL-1β*, and *leptin*. Serum levels of IL-6, IL-1β, leptin, and insulin were also significantly reduced but the changes in insulin levels are of minor clinical significance. As the changes in blood mRNA gene expressions of interested outcomes were with high effect sizes, these findings suggest that NS oil can be considered a beneficial supplemental therapy to help prevent and manage overweight and obesity, and reduce the burden of the disease in the general population. However, further interventions are required to confirm the carryover effects observed for leptin concentration and the small effect size estimated in serum insulin changes. Future studies investigating the effects of NS supplements on various pro-inflammatory markers in different genders and populations may help improve the generalizability of these findings. The use of the next-generation sequencing (NGS) method in future gene-diet interaction investigations is strongly recommended prior to assessing gene expression to have the most comprehensive genomic coverage and to more accurately detect variations of significance.

### Electronic supplementary material

Below is the link to the electronic supplementary material.

Additional file 1

Additional file 2

## Data Availability

Data are available from the correspondence author upon reasonable request and with permission of Shahid Sadoughi Medical University.

## List of abbreviations

BMI: Body mass index
cDNA: Complementary DNA
CI: Confidence intervals
CONSORT: Consolidated Standards of Reporting Trials
Ct: Cycle threshold
d: Cohen’s d
ELISA: Enzyme-linked immunoassay
FD: Fold changes
GAPDH: Glyceraldehyde phosphate dehydrogenase
GC/MS: Gas chromatography-mass spectrometry
IL-1β: Interleukin 1 beta
IL-6: Interleukin six
ITT: The intention-to-treat
MAPK: Mitogen-activated protein kinase
MET: Metabolic equivalent
NF-κB: Nuclear factor kappa B
NS: Nigella sativa
PBMC: Peripheral blood mononuclear cells
RCT: Randomized controlled trial
RNA: Ribonucleic acid
RT-PCR: Real-time polymerase chain reaction
TQ: Thymoquinone
