# Supplementary material for: A crossover randomized controlled trial examining the effects of black seed (Nigella sativa) supplementation on IL-1β, IL-6 and leptin, and insulin parameters in overweight and obese women
Source: BMC Complement Med Ther. 2024 Jan 5;24:22. doi: 10.1186/s12906-023-04226-y (PMC10768077; doi:10.1186/s12906-023-04226-y)
Supplement: Supplementary file 1 — Additional file 1 [file 12906_2023_4226_MOESM1_ESM.pdf]

Supplementary file 1: GC/MS a test of each 50 g of *Nigella sativa* powder

**Test**	**Reference method**	**Acceptable value**	**Result**
Appearance	Visual	Brown color	Brown color
Odor	Organoleptic	Specific	Specific
Palmitic acid (%)	WR10[b]	11–14	11.84
Stearic acid (%)	WR10	–	2.00
Oleic acid (%)	WR10	17–26	22.14
Linoleic acid (%)	WR10	52067	59.66
Linolenic acid (%)	WR10	–	1.79
Thymoquinone (%)	EP[c] 0.8 (2.2.23)	–	0.54

[a] Gas chromatography–mass spectrometry

[b] Rectangular waveguide size

[c] European Pharmacopoeia
