# Supplementary material for: A crossover randomized controlled trial examining the effects of black seed (Nigella sativa) supplementation on IL-1β, IL-6 and leptin, and insulin parameters in overweight and obese women
Source: BMC Complement Med Ther. 2024 Jan 5;24:22. doi: 10.1186/s12906-023-04226-y (PMC10768077; doi:10.1186/s12906-023-04226-y)
Supplement: Supplementary file 2 — Additional file 2 [file 12906_2023_4226_MOESM2_ESM.pdf]

A

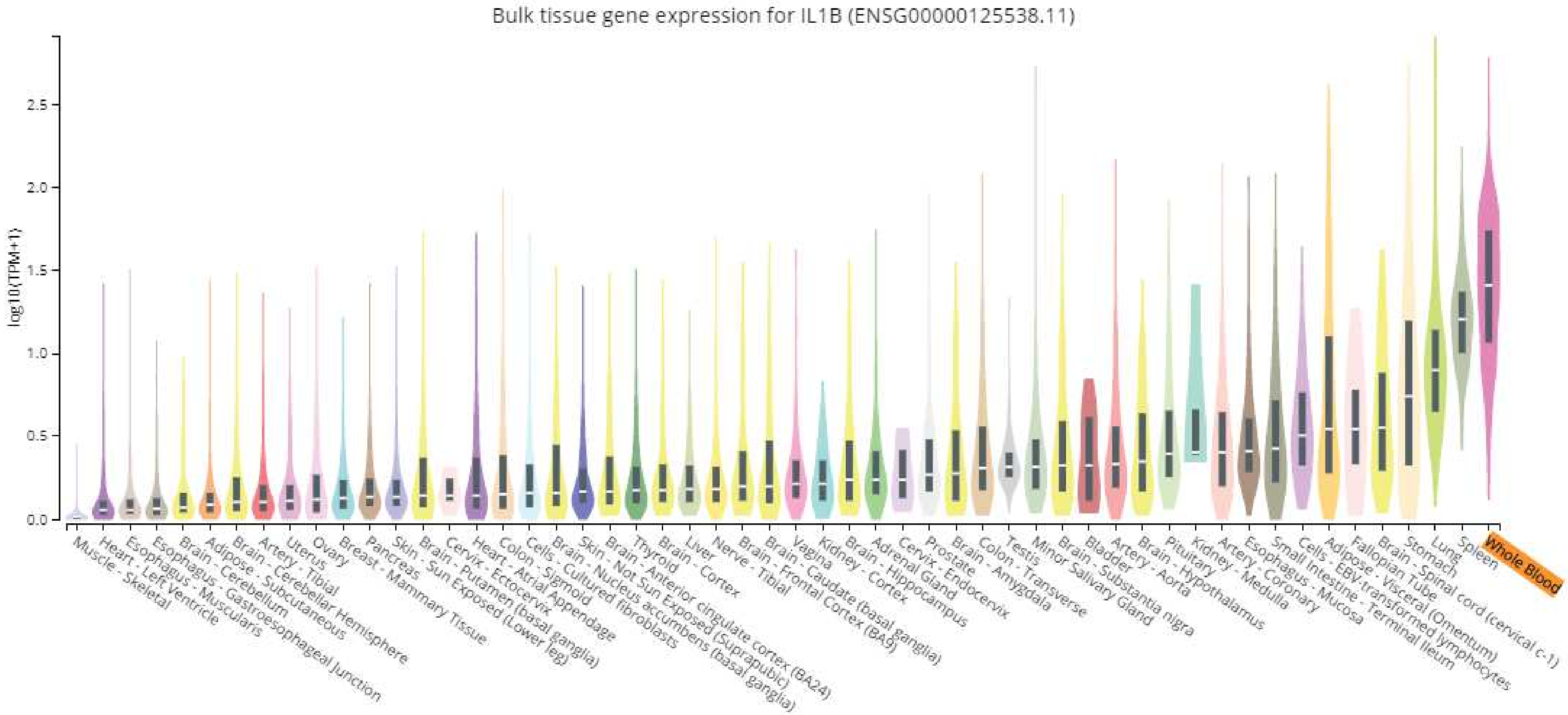
Bulk tissue gene expression for IL1B (ENSG00000125538.11)
log10(TPM+1)
0.0
0.5
1.0
1.5
2.0
2.5
Muscle - Skeletal
Heart - Left Ventricle
Esophagus - Muscularis
Esophagus - Gastroesophageal Junction
Brain - Cerebellum
Adipose - Subcutaneous
Brain - Cerebellar Hemisphere
Artery - Tibial
Uterus
Ovary
Breast - Mammary Tissue
Pancreas
Skin - Sun Exposed (Lower leg)
Brain - Putamen (basal ganglia)
Cervix - Ectocervix
Heart - Atrial Appendage
Colon - Sigmoid
Cells - Cultured fibroblasts
Brain - Nucleus accumbens (basal ganglia)
Skin - Not Sun Exposed (Suprapubic)
Brain - Anterior cingulate cortex (BA24)
Thyroid
Brain - Cortex
Liver
Nerve - Tibial
Brain - Frontal Cortex (BA9)
Brain - Caudate (basal ganglia)
Vagina
Kidney - Cortex
Brain - Hippocampus
Adrenal Gland
Cervix - Endocervix
Prostate
Brain - Amygdala
Colon - Transverse
Testis
Minor Salivary Gland
Brain - Substantia nigra
Bladder
Artery - Aorta
Brain - Hypothalamus
Pituitary
Kidney - Medulla
Artery - Coronary
Esophagus - Mucosa
Small Intestine - Terminal Ileum
Cells - EBV-transformed lymphocytes
Adipose - Visceral (Omentum)
Fallopian Tube
Brain - Spinal cord (cervical c-1)
Stomach
Lung
Spleen
Whole Blood

B

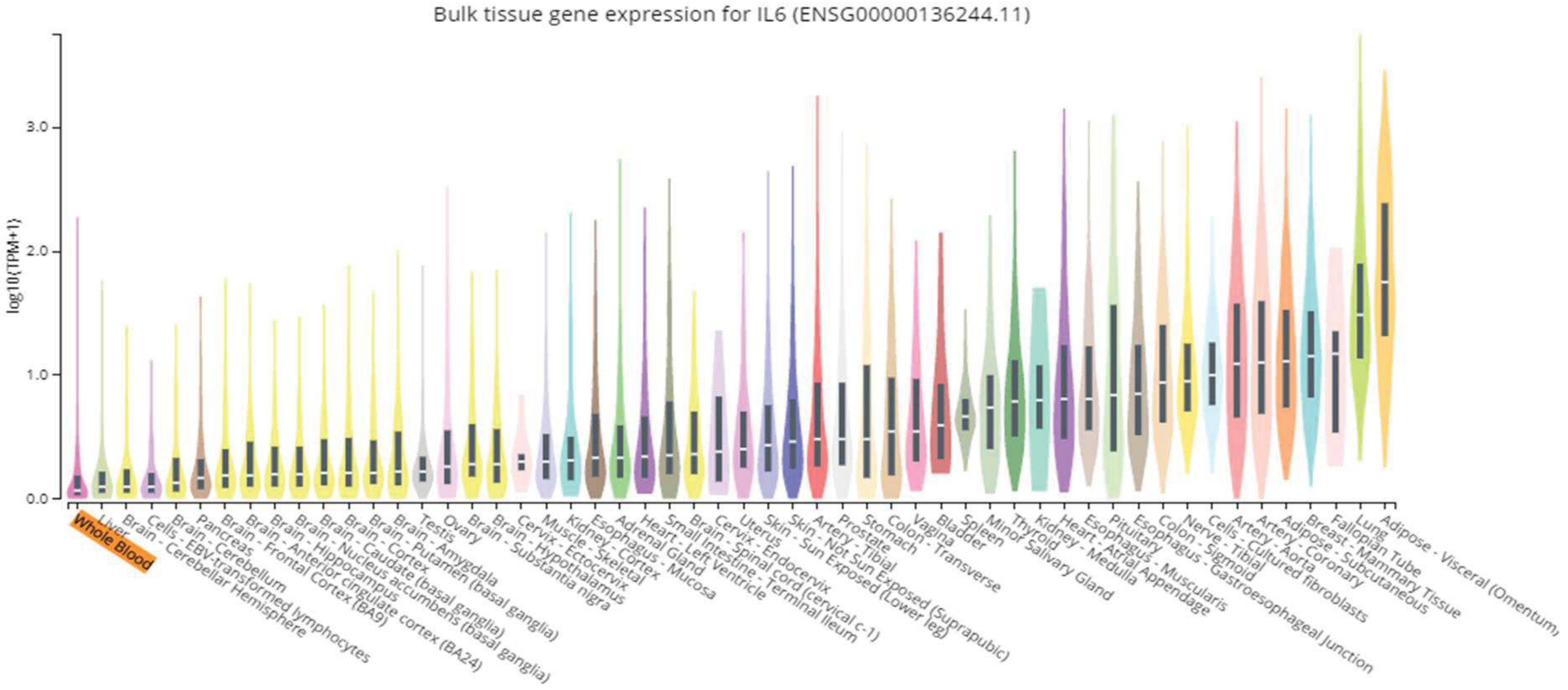
Bulk tissue gene expression for IL6 (ENSG00000136244.11)
log10(TPM+1)
0.0
1.0
2.0
3.0
Whole Blood
Liver
Brain - Cerebellar Hemisphere
Cells - EBV-transformed lymphocytes
Brain - Cerebellum
Pancreas
Brain - Frontal Cortex (BA9)
Brain - Anterior cingulate cortex (BA24)
Brain - Hippocampus
Brain - Nucleus accumbens (basal ganglia)
Brain - Caudate (basal ganglia)
Brain - Cortex
Brain - Putamen (basal ganglia)
Brain - Amygdala
Testis
Ovary
Brain - Substantia nigra
Brain - Hypothalamus
Cervix - Ectocervix
Muscle - Skeletal
Kidney - Cortex
Esophagus - Mucosa
Adrenal Gland
Heart - Left Ventricle
Small Intestine - Terminal Ileum
Brain - Spinal cord (cervical c-1)
Cervix - Endocervix
Uterus
Skin - Sun Exposed (Lower leg)
Skin - Not Sun Exposed (Suprapubic)
Artery - Tibial
Prostate
Stomach
Colon - Transverse
Vagina
Bladder
Spleen
Minor Salivary Gland
Thyroid
Kidney - Medulla
Heart - Atrial Appendage
Esophagus - Muscularis
Pituitary
Esophagus - Gastroesophageal Junction
Colon - Sigmoid
Nerve - Tibial
Cells - Cultured fibroblasts
Artery - Aorta
Artery - Coronary
Adipose - Subcutaneous
Breast - Mammary Tissue
Fallopian Tube
Lung
Adipose - Visceral (Omentum)

C

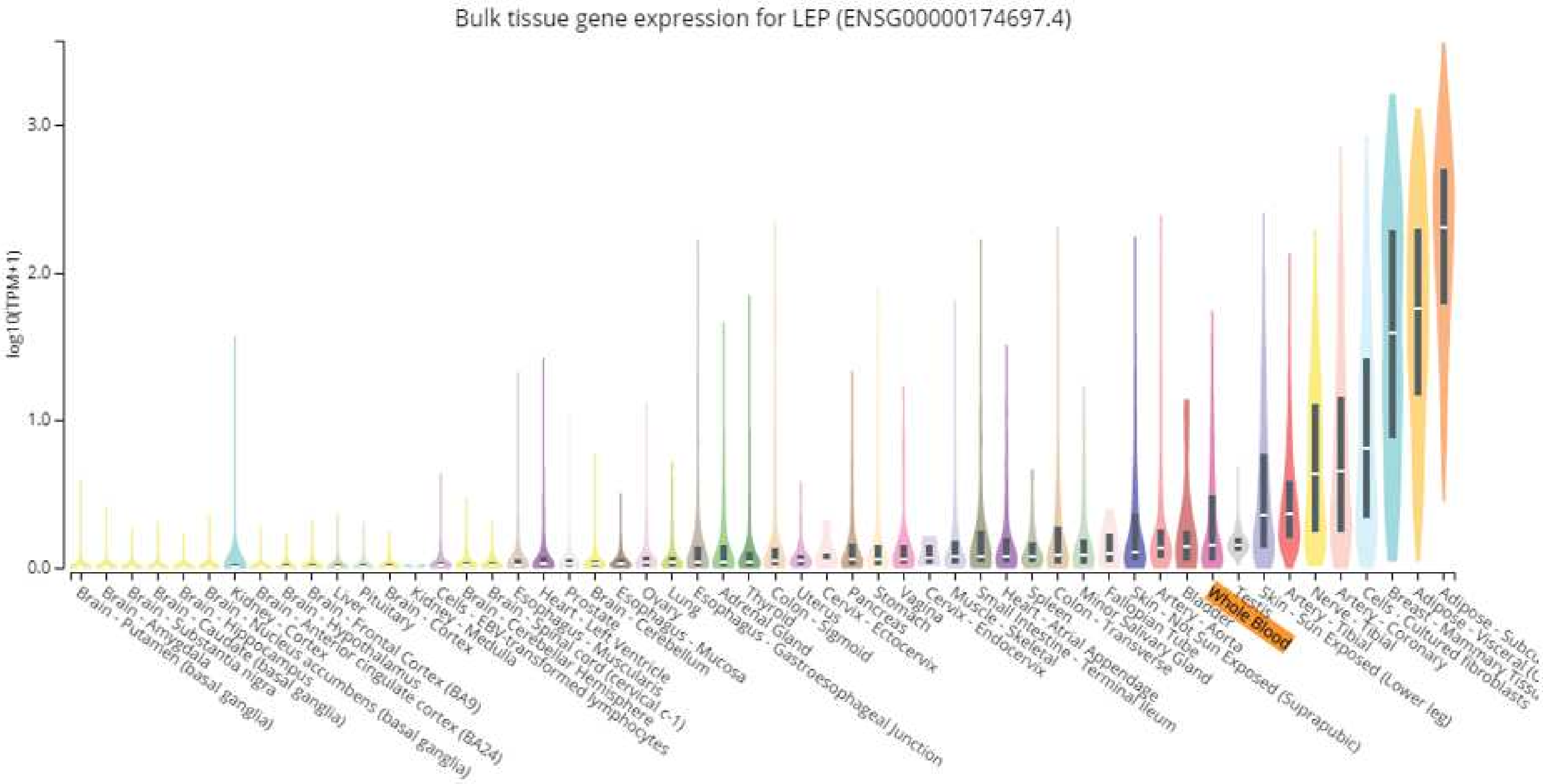

Supplementary file 2: Based on the GTEx portal, **A** IL-1β, **B** IL-6 and **C** Leptin have been identified in blood by RNA sequencing.
